# Food Industry By-Products as Raw Materials in the Production of Value-Added Corn Snack Products

**DOI:** 10.3390/foods10050946

**Published:** 2021-04-26

**Authors:** Antun Jozinović, Drago Šubarić, Đurđica Ačkar, Jurislav Babić, Vedran Orkić, Sunčica Guberac, Borislav Miličević

**Affiliations:** 1 Faculty of Food Technology Osijek, Josip Juraj Strossmayer University of Osijek, Franje Kuhača 18, 31000 Osijek, Croatia; drago.subaric@ptfos.hr (D.Š.); dackar@ptfos.hr (Đ.A.); jurislav.babic@ptfos.hr (J.B.); borislav.milicevic@ptfos.hr (B.M.); 2 Scientific Center of Excellence for Personalized Health Care, Josip Juraj Strossmayer University of Osijek, Trg sv. Trojstva 3, 31000 Osijek, Croatia; 3 Faculty of Agrobiotechnical Sciences Osijek, Josip Juraj Strossmayer University of Osijek, Vladimira Preloga 1, 31000 Osijek, Croatia; vedran.orkic@fazos.hr (V.O.); suncica.guberac@fazos.hr (S.G.); 4 Department of Agriculture, Polytechnic in Požega, Vukovarska 17, 34000 Požega, Croatia

**Keywords:** by-products, extrusion, corn snacks, chemical composition, nutritional value

## Abstract

The addition of brewer’s spent grain (BSG), sugar beet pulp (SBP) and apple pomace (AP), on the nutritional properties of directly expanded snack products based on corn grits was investigated. Snack products were produced in a laboratory single screw extruder with the addition of 5, 10 and 15% d. m. of these by-products in corn grits. Chemical composition, total phenolic content, antioxidant activity, dietary fiber, resistant starch, starch damage and pasting properties of the mixtures and extruded samples were determined. Extrusion process and by-product additions had a significant effect on the proximate chemical composition. All three by-products increased contents of both soluble and insoluble dietary fiber, while the extrusion caused a reduction of insoluble fiber and increase of soluble fiber. After the extrusion process starch damage and antioxidant activity increased, while resistant starch content and total polyphenol content decreased. According to obtained results, it can be concluded that the investigated by-products can be used in the production of nutritionally more valuable corn snacks.

## 1. Introduction

The development of value-added foods in terms of maintaining and protecting health, improving consumer nutrition and promoting good health and nutrition has been prioritized by numerous researches in recent years. Since the United Nations published Sustainable Development Goals [1], which has been adopted in strategies worldwide, both science and industry have been challenged to look for solutions to utilize by-products of the food industry in such a way that they are first used as food, then as feed and finally for energy production. These by-products are of particular interest because they often contain significantly high amounts of certain compounds (bioactive components, dietary fibers, vitamins, minerals, etc.), which constitute a very valuable raw material for the production and development of new products [2,3,4]. One of the most important products are directly expanded cereal-based snacks, which are currently high in carbohydrates, salt and fat and low in overall nutritional value, popular and consumed in large quantities by all generations, from babies to the elderly [5,6,7].

Although numerous studies have been conducted on the use of by-products as cheap and high-value raw materials in the development of new extruded products, most of them are focused on testing the formulations but not the overall sensory and nutritional quality of the developed products [8]. In this context, in our previous studies [9,10] we investigated the physical and sensory properties of extruded products with added brewer’s spent grain (BSG), sugar beet pulp (SBP) and apple pomace (AP) in order to obtain products acceptable for consumption. In first study we investigated influence of by-products addition on physical and sensory properties of corn extrudates, while in the second one, a safety of these products was carried out, manifested by the formation of potentially harmful components (acrylamide and hydroxymethylfurfural). The composition of all three by-products used in this study was presented in our previous research [9] and in other reviews [2,4,8]. AP was used in the production of extruded snacks based on sorghum flour [11] or rice-wheat mixture [12] and the extrudates based on pregelatinized starch combined with cheese whey or other protein sources [6]. BSG was used in content of 10% for the production of barley-based snacks [13] and in the production of extruded broken rice snacks with 15% and 30% of BSG [14]. SBP as a by-product is mainly used for animal feeding, but as a good source of pectin this by-product is recently used for the pectin extraction although pectin extracted from SBP is known to have poor gelling property [15].

The aim of this study was to investigate how the addition of the above-mentioned by-products affect the nutritional value of the developed corn snack products in terms of chemical composition, total phenolic content, antioxidant activity, dietary fiber, resistant starch and starch damage.

## 2. Materials and Methods

### 2.1. Preparation of Extruded Corn Snack Products

Raw materials used in this study were: corn grits, brewer’s spent grain (BSG), sugar beet pulp (SBP), apple pomace (AP) and pectin (GENU^®^ Pectin 150 USA-SAG type D slow set, CP Kelco A Huber Company, SAD). The chemical composition of raw materials and the procedure of the blend preparation for the extrusion process were described in detailed in our previous article [9]. Briefly, BSG, SBP and AP were added in corn grits in the content of 5%, 10% and 15% d. m. In the case of BSG and SBP, samples with 1% of added pectin were prepared, which resolved the problem of expansion and poor texture properties and enabled the production of sensory acceptable expanded snack products [9].

The blends with 15% moisture content were extruded in a laboratory single-screw extruder (Brabender GmbH, Model 19/20DN, Duisburg, Germany) according to Ačkar et al. [9], at the temperature profile: 135/170/170 °C, using a screw with a compression ratio of 4:1, and a round die head with 4 mm nozzle diameter. The obtained snacks were air-dried overnight at ambient temperature, milled in a laboratory mill (IKA MF10, Staufen, Germany) with a 1 mm sieve and stored in sealed plastic bags at 4 °C until further analysis.

### 2.2. Chemical Composition

The chemical composition of non-extruded samples and obtained extrudates were determined according to standard methods for moisture content (ISO 6540) [16], protein (ISO 5983-2) [17], fat (ISO 6492) [18] and ash (ISO 5984) [19], while the contents of raw carbohydrates were calculated by difference [9].

### 2.3. Dietary Fiber

Total, soluble and insoluble dietary fiber (TDF, SDF and IDF) were determined according to the AOAC 991.43 method [20], using K-TDFR kit (Megazyme Int. Wicklow, Ireland).

### 2.4. Resistant Starch, Starch Damage and Pasting Properties

Resistant starch (RS) was determined according to the AOAC 2002.02 method [21], and starch damage (DS) according to the AACC 76-31.01 method [22], using K-RSTAR and K-SDAM kits (Megazyme Int. Wicklow, Ireland), respectively. Pasting properties (peak, hot and cold viscosities) of non-extruded and extruded samples were measured according to Jozinović et al. [23] using a Micro Visco-Analyser (Brabender Gmbh & Co KG, Duisburg, Germany).

### 2.5. Total Phenolic Content and DPPH Radical Scavenging Activity

The total phenolic content (TPC) of the sample extracts in methanol:water (80:20) was determined by the Folin–Ciocalteu colorimetric method, while the DPPH radical scavenging activity of the extracts was estimated using the stable DPPH radical. Both methods are described in detail by Wang and Ryu [24].

### 2.6. Statistical Analysis

The obtained results based on the statistical calculations from three parallel repetitions for all analysis were analyzed using analysis of variance (ANOVA) and Duncan’s least significant difference (LSD) test, with the significant difference of the data at *p* < 0.05 in the software STATISTICA 13.3 (StatSoft, Inc., Tulsa, OK, USA) and Microsoft Office Excel 2019.

## 3. Results and Discussion

### 3.1. Chemical Composition

Brewer’s spent grain (BSG), sugar beet pulp (SBP) and apple pomace (AP) as food industry by-products used in this study for the development of new value-added corn snack products represent a great potential due to their chemical composition presented in our previous article [9]. The influence of the addition of BSG, SBP and AP in corn grits and the effect of the extrusion process on the chemical composition is shown in Table 1.

Increasing the proportion of BSG and SBP in the mixtures, the protein content increased, and the increase was more pronounced with the addition of BSG. Thus, the protein content in the mixture with 15% BSG was 11.08% ± 0.14% d. m., and in the mixture with 15% SBP was 8.08% ± 0.01% d. m., compared to the corn grits, in which the protein content was 7.91% ± 0.05% d. m. On the other hand, the addition of AP to the mixture proportionally reduced the protein content, so the lowest protein content was recorded in the mixture containing 15% AP (7.07% ± 0.05% d. m.). The fat content decreased with the addition of AP and SBP to the mixture, while the increase in fat content was recorded in the mixtures with the addition of BSG. The lowest fat content was in the sample with 15% SBP (0.99% ± 0.01% d. m.), and the highest in the sample with 15% BSG (1.91% ± 0.02% d. m.). The ash content was increased by the addition of all by-products in proportion to the added content, with the highest value recorded for the mixture containing 15% SBP (1.64% ± 0.01% d. m.). The content of raw carbohydrates decreased with the addition of BSG and SBP to corn grits, while the opposite trend was observed in mixtures with AP, so the mixture with 15% AP had the highest content of raw carbohydrates (91.31% ± 0.03% d. m.).

From the results obtained for the extruded samples, it is visible that the extrusion process resulted in a decrease in the values of protein and fat content for all samples, while the ash content increased, but this increase was not so high. This trend of decreasing protein and fat content and not so high increase in ash content after the extrusion process is consistent with previous studies. In short, the most significant change in proteins during the extrusion process is denaturation due to the applied high temperatures, pressure and shear [25,26,27]. Sobota et al. [25] concluded that the extrusion process resulted in a decrease of protein content, which is in accordance with the results of our study. The lack of nitrogen content due to the extrusion process is explained by Stanley [28] as the effect of the formation of isopeptide bonds between the ε-amine group of lysine and the amide groups of asparagines or glutamine, which is associated with ammonia release. Furthermore, the reaction of lysine with reducing sugars formed during the extrusion is also stated [29]. As for fats, their proportion is reduced mainly by loss at the die after leaving the extruder and by the formation of fat complexes with starch and proteins [29,30]. In agreement with the effect of extrusion on the reduction of protein and fat content, the content of raw carbohydrates increased in all samples after the extrusion process, so that the highest content was recorded in the extruded sample with 15% AP (91.88% ± 0.02% d. m.). The results obtained in this research are in accordance with previous studies, where it was found that the content of protein, fat and ash in extruded products increases with the addition of used by-products [6,31,32,33].

### 3.2. Dietary Fiber

The by-products used to fortify corn snack products in this study were selected in part because of the high fiber content found in the literature [34,35,36], and confirmed by the results shown in our previous study [9]. Namely, all by-products had significantly higher content of total, soluble and insoluble dietary fiber (TDF, SDF and IDF), compared to the corn grits. While corn grits contained 3.39% ± 0.01% d. m. of TDF, this content was >10 times higher in AP (40.47% ± 0.26% d. m.), >15 times higher in BSG (60.56% ± 0.53% d. m.) and >20 times higher in SBP (70.98% ± 0.29% d. m.) [9]. The results for dietary fiber content in non-extruded mixtures and produced snack products are shown in the Table 2. The obtained results show that by increasing the amount of added by-products in the mixtures, the content of IDF and SDF increased proportionally, with BSG having the most significant influence on the increase of IDF content (11.22% ± 0.07% d. m., for the mixture with 15% of BSG), and SBP on the SDF content (3.45% ± 0.08% d. m., for the mixture with 15% SBP). Despite the fact that the addition of AP increased the fiber content less than other by-products, the values obtained were significantly higher compared to corn grits, which is confirmed by the fact that the TDF content doubled already with the addition of 10% AP. The effect of higher fiber increment in the case of SBP and BSG is also related to the fact that in the application of these two by-products also 1% d. m. of pectin was added, which belongs to the group of SDF.

The extrusion process had the same effect on all samples, regardless of the type of by-product used. Namely, a decrease in the IDF content and an increase in the SDF content was found. Since the decrease in the IDF content was more pronounced than the increase in the SDF content in all cases, this also affected the decrease in the TDF content in all extrudates. However, it should be emphasized that even after these changes due to the extrusion process, the obtained extrudates with satisfactory expansion and sensory acceptability had a significantly higher content of dietary fiber compared to the control sample of extruded corn grits. Namely, while the extruded sample of corn grits had 2.36% ± 0.03% d. m. of TDF content, extrudates with BSG and SBP had double the amount of TDF content already at 5% added by-products (5.13% ± 0.08% d. m. and 5.56% ± 0.21% d. m., respectively), and a similar trend was observed with the addition of AP, where the sample with 5% AP had 4.57% ± 0.05% d. m. of TDF content.

Previous studies have shown that the extrusion process generally reduces the content of IDF, with the usual accompanying increase in the content of SDF, which would imply that a conversion from insoluble to soluble fibers occurs during the extrusion process [24,37,38]. Such results have been obtained by Sobota et al. [25], Pérez-Navarrete et al. [39] and Jing and Chi [26], which particularly point out the increase in IDF content after the extrusion process. Therefore, the results of this research are in accordance with all the above studies. Furthermore, in terms of the impact of the by-products used in this study on the increase in fiber content, the obtained results are also consistent with previous studies. Namely, the addition of BSG increased the dietary fiber content in the production of wheat bread [40] and in various extruded products [13,31,32,41,42]. So, Kirjoranta et al. [13] used 10% of BSG in the production of barley based snacks and concluded that obtained extruded were “high fiber”. Similarly, the “high fiber” extrudates were developed with application of 20–40% BSG in extruded products based on rice flour and wheat semolina mixture [42]. The use of sugar beet fiber increased the content of dietary fiber in spaghetti production [43] and corn extrudates [44]. The same effect has also been reported with the use of AP in cake making [45] and in corn extrudates [46]. Additionally, the results obtained in our research are in agreement with the results obtained in the preparation of rice-wheat based extruded samples with AP (10–30%), where it was found that the addition of AP increased the TDF content [12]. In addition, incorporation of 22% AP in snacks based on pregelatinized starch the dietary fiber content increased from 0.8 g/100 g in the control sample to 14 g/100 g in the sample containing AP [6].

According to all of the above, it is important to point out that the obtained extrudates with 5% of added by-products may carry nutrition claim “source of fiber” according to Regulation (EU) No 1924/2006 and Regulation (EC) No 1047/2012 [47], as the dietary fiber content in all samples exceeded 3 g/100 g, while the extrudates with 10% and 15% of added by-products may carry nutrition claim “high in fiber”, since dietary fiber content in these samples was above 6 g/100 g.

### 3.3. Resistant Starch, Starch Damage and Paste Properties 

The influence of the addition of BSG, SBP and AP in corn grits and the effect of the extrusion process on the content of resistant starch (RS) and non-resistant starch (NRS) and on starch damage (DS) and pasting properties is shown in Table 3.

The obtained results show that in the mixtures with added by-products, the content of NRS decreased proportionally to the amount of added by-products, and the effect was most pronounced in the case of AP addition. On the other hand, the same trend of decrease in RS content was maintained in the case of SBP and AP, while with the addition of BSG in corn grits the content of RS increased. As for the influence of the extrusion process, it resulted in a significant decrease in RS content and an increase in NRS content in all samples, regardless of the added by-product. All of these is consistent with the well-known changes that take place during the extrusion process. Indeed, during the process, the combined effect of high shear, high pressure and high temperature leads to gelatinization and depolymerization of the starch molecules [29,48,49], making them more susceptible to attack by enzymes. This is confirmed by the results of previous studies, both ours [23,50] and those of other researchers [31,39,48,51]. The same tendency of the effect of extrusion process on increasing starch digestibility was observed in the preparation of sorghum-based extrudates with the addition of AP [11] and in the rice-wheat-based snacks with BSG [42], confirming the results of this study. Significant changes in starch after the extrusion process were confirmed by the results obtained for the percentage of DS, which increased significantly in the extruded samples. Additionally, the results obtained for the pasting properties for peak, hot and cold viscosity values confirmed that the extrusion process caused significant increase in starch damage. Indeed, it is well known that the decrease in peak viscosity is associated with greater degradation and gelatinization of starch during the extrusion process [23,52].

### 3.4. Total Phenolic Content and DPPH Radical Scavenging Activity

In addition to dietary fiber, this study also investigated the effect of adding by-products on polyphenol content and antioxidant activity, as another aspect to increase the nutritional value of extruded snacks. Results for total polyphenol content (TPC) and antioxidant activity (AOA) are shown in Table 4.

While the addition of SBP to the mixture leads to a decrease in TPC content and AOA, on the other hand the addition of BSG showed an increase in TPC, but a decrease in AOA. However, this effect on TPC and AOA is not so significantly pronounced in the case of BSG and SBP addition, as it is with the application of AP. Thus, the highest values for the proportion of TPC in the mixture were obtained for mixtures containing 15% AP (421.09 ± 3.58 mg GAL/100 g d. m.) and for AOA (38.31 ± 0.27% DPPH), compared to the corn grits (61.38 ± 1.64 mg GAL/100 g d. m. and 17.78 ± 0.03 % DPPH, respectively). These results are consistent with previously published reviews [2,4] showing that polyphenols are predominantly localized in the peels and the main compounds isolated and identified in AP include catechins, hydroxycinnamates, phloretin glycosides, quercetin glycosides, procyanidins, chlorogenic and caffeic acids and phloridzin. After the extrusion process, a decrease in TPC content was observed in all samples, while on the other hand extrusion had the opposite effect on AOA, which increased in all snack products.

From the obtained results for TPC and AOA in extrudates, it can be seen that the lowest value of TPC had an extruded sample of corn grits (48.39 ± 1.06 mg GAL/100 g d. m.), and the highest had a sample with 15% AP (409.13 ± 6.03 mg GAL/100 g d. m.). The lowest value of AOA was recorded in extrudates containing 15% SBP (16.64% ± 0.61% DPPH), and the highest again in the sample containing 15% AP (78.11% ± 1.08% DPPH). The fact that the polyphenol content decreases during the extrusion process has been found in other studies [24,53,54], which is in accordance with the results obtained in this study. Sharma et al. [55] state that the decrease in polyphenol content after the extrusion process may be the result of their decomposition at high extrusion temperatures or a change in the structure that causes reduced reactivity of phenolic compounds. Regarding the increase in antioxidant after the extrusion process, this could be explained by the results obtained for the content of acrylamide and hydroxymethylfurfural presented in our previously published work [10]. Namely, during the extrusion process, these undesirable products of Maillard reactions are formed, which have been found to also exhibit antioxidant activity [56], and therefore antioxidant activity in extruded products could be affected not only by polyphenols but also by the compounds formed [12]. Thus, the greatest increase in AOA after extrusion was shown by the sample containing 15% AP (38.31% ± 0.27% DPPH in the mixture; 78.11% ± 1.08% DPPH in the extrudate), which is in accordance with the highest obtained results for the content of acrylamide (5.37 ± 0.50 ng/g) and hydroxymethylfurfural (6068.52 ± 788.80 ng/g) for the extruded sample with 15% AP, reported in our previous research [10]. Finally, it is important to emphasize that the obtained snack products had higher polyphenol content compared to the control sample, and the effect was most pronounced in the samples with AP. This is confirmed by the results of other research. So, the addition of BSG increased TDF and AOA in the extrudates based on rice flour and wheat semolina [42]. Furthermore, the extrudates with 30% AP had higher TPC and AOA compared to the control sample based on sorghum flour [11]. The same tendency was observed in the rice-wheat based extrudates, but it was concluded that the recoveries of phenolic compounds decreased with addition of higher content of AP due to polymerization during extrusion process [12].

## 4. Conclusions

The addition of all three by-products and the extrusion process had a significant effect on the proximate chemical composition, with the most pronounced effect on protein content in the case of BSG addition. The content of both soluble and insoluble dietary fiber was increased with addition of all by-products, while extrusion caused a reduction of insoluble fiber and increase of soluble fiber. According to the results for total dietary fiber content it is important to emphasize that the obtained extrudates with 5% of added by-products may carry a nutrition claim “source of fiber”, as the dietary fiber content in all samples exceeded 3 g/100 g, while the extrudates with 10% and 15% of added by-products may carry a nutrition claim “high in fiber”, since dietary fiber content in these samples was above 6 g/100 g. Application of SBP had the best effect on nutritional value from the view of dietary fiber content. The extrusion process caused an increase in starch damage and antioxidant activity, while resistant starch content and total polyphenol content decreased. The most pronounced effect on the polyphenol content and antioxidant activity was observed by the application of AP. According to all of the above, it can be concluded that the addition of investigated by-products can significantly improve the nutritional value of corn snack products in terms of increasing the amount of dietary fiber, total polyphenol content and antioxidant activity.

## Figures and Tables

**Table 1:** Chemical composition of non-extruded and extruded samples.

**Sample**	**NON-EXTRUDED**				
	**Dry Matter** **(%)**	**Protein** **(% d. m.)**	**Fat** **(% d. m.)**	**Ash** **(% d. m.)**	**Raw Carbohydrates** **(% d. m.)**
Corn grits	85.25 ± 0.04 ^b^	7.91 ± 0.05 ^d^	1.33 ± 0.02 ^e^	0.41 ± 0.02 ^a^	90.35 ± 0.05 ^f^
Corn + 5% BSG	85.11 ± 0.02 ^a^	8.94 ± 0.06 ^f^	1.52 ± 0.03 ^g^	0.67 ± 0.01 ^e^	88.87 ± 0.11 ^c^
Corn + 10% BSG	85.78 ± 0.03 ^e^	9.68 ± 0.07 ^g^	1.47 ± 0.00 ^f^	0.77 ± 0.01 ^f^	88.08 ± 0.08 ^b^
Corn + 15% BSG	85.39 ± 0.06 ^c^	11.08 ± 0.14 ^h^	1.91 ± 0.02 ^h^	0.93 ± 0.01 ^h^	86.08 ± 0.11 ^a^
Corn + 5% SBP	85.19 ± 0.02 ^b^	7.80 ± 0.10 ^d^	1.18 ± 0.01 ^d^	0.81 ± 0.03 ^g^	90.21 ± 0.12 ^f^
Corn + 10% SBP	85.05 ± 0.02 ^a^	7.83 ± 0.14 ^d^	1.04 ± 0.01 ^b, c^	1.30 ± 0.02 ^i^	89.83 ± 0.18 ^e^
Corn + 15% SBP	85.20 ± 0.05 ^b^	8.08 ± 0.01 ^e^	0.99 ± 0.01 ^a^	1.64 ± 0.01 ^j^	89.29 ± 0.03 ^d^
Corn + 5% AP	85.57 ± 0.03 ^d^	7.57 ± 0.05 ^c^	1.18 ± 0.01 ^d^	0.46 ± 0.00 ^b^	90.79 ± 0.07 ^g^
Corn + 10% AP	85.35 ± 0.00 ^c^	7.30 ± 0.09 ^b^	1.07 ± 0.03 ^c^	0.53 ± 0.03 ^c^	91.10 ± 0.08 ^h^
Corn + 15% AP	85.42 ± 0.02 ^c^	7.07 ± 0.05 ^a^	1.00 ± 0.01 ^a,b^	0.62 ± 0.02 ^d^	91.31 ± 0.03 ^i^
**Sample**	**EXTRUDED**				
	**Dry Matter** **(%)**	**Protein** **(% d. m.)**	**Fat** **(% d. m.)**	**Ash** **(% d. m.)**	**Raw Carbohydrates** **(% d. m.)**
Corn grits	91.69 ± 0.01 ^a^	7.84 ± 0.03 ^d^	0.23 ± 0.02 ^a^	0.45 ± 0.00 ^a^	91.48 ± 0.02 ^g^
Corn + 5% BSG	91.86 ± 0.02 ^b^	8.72 ± 0.01 ^e^	0.43 ± 0.00 ^c,d^	0.70 ± 0.00 ^d^	90.15 ± 0.00 ^d^
Corn + 10% BSG	92.21 ± 0.01 ^c^	9.58 ± 0.03 ^f^	0.52 ± 0.03 ^e, f^	0.77 ± 0.01 ^e^	89.13 ± 0.07 ^b^
Corn + 15% BSG	92.77 ± 0.00 ^g^	10.88 ± 0.09 ^g^	1.23 ± 0.04 ^g^	0.96 ± 0.02 ^g^	86.93 ± 0.14 ^a^
Corn + 5% SBP	91.88 ± 0.01 ^b^	7.78 ± 0.03 ^d^	0.42 ± 0.00 ^b,c^	0.87 ± 0.02 ^f^	90.93 ± 0.01 ^f^
Corn + 10% SBP	92.65 ± 0.00 ^e^	7.79 ± 0.03 ^d^	0.38 ± 0.06 ^b,c^	1.36 ± 0.02 ^h^	90.47 ± 0.05 ^e^
Corn + 15% SBP	92.69 ± 0.02 ^f^	7.85 ± 0.04 ^d^	0.57 ± 0.05 ^f^	1.78 ± 0.02 ^i^	89.80 ± 0.08 ^c^
Corn + 5% AP	92.40 ± 0.00 ^d^	7.42 ± 0.04 ^c^	0.34 ± 0.05 ^b^	0.54 ± 0.04 ^b^	91.70 ± 0.05 ^h^
Corn + 10% AP	92.63 ± 0.01 ^e^	7.24 ± 0.01 ^b^	0.44 ± 0.01 ^c–e^	0.59 ± 0.03 ^c^	91.73 ± 0.05 ^h^
Corn + 15% AP	92.76 ± 0.01 ^g^	6.95 ± 0.05 ^a^	0.51 ± 0.02 ^d–f^	0.66 ± 0.01 ^d^	91.88 ± 0.02 ^i^

Values with different letters in the same column and group of samples (non-extruded and extruded) are significantly different at *p* < 0.05. BSG—brewer’s spent grain, SBP—sugar beet pulp, AP—apple pomace. In the samples with BSG and SBP 1% d. m. of pectin was added.

**Table 2:** The dietary fiber content in non-extruded and extruded samples.

**Sample**	**NON-EXTRUDED**		
	**IDF** **(% d. m.)**	**SDF** **(% d. m.)**	**TDF** **(% d. m.)**
Corn grits	3.18 ± 0.03 ^a^	0.21 ± 0.04 ^a^	3.39 ± 0.01 ^a^
Corn + 5% BSG	5.98 ± 0.05 ^d^	1.29 ± 0.09 ^c^	7.26 ± 0.04 ^d^
Corn + 10% BSG	8.72 ± 0.07 ^g^	1.44 ± 0.12 ^c^	10.16 ± 0.20 ^g^
Corn + 15% BSG	11.22 ± 0.07 ^i^	1.64 ± 0.06 ^d^	12.86 ± 0.01 ^i^
Corn + 5% SBP	5.69 ± 0.17 ^c^	1.97 ± 0.11 ^e^	7.66 ± 0.06 ^e^
Corn + 10% SBP	8.34 ± 0.10 ^f^	2.51 ± 0.03 ^f^	10.85 ± 0.13 ^h^
Corn + 15% SBP	10.70 ± 0.10 ^h^	3.45 ± 0.08 ^g^	14.15 ± 0.18 ^j^
Corn + 5% AP	4.44 ± 0.07 ^b^	0.75 ± 0.09 ^b^	5.19 ± 0.02 ^b^
Corn + 10% AP	5.62 ± 0.07 ^c^	1.29 ± 0.02 ^c^	6.91 ± 0.08 ^c^
Corn + 15% AP	6.89 ± 0.05 ^e^	1.92 ± 0.02 ^e^	8.80 ± 0.07 ^f^
**Sample**	**EXTRUDED**		
	**IDF** **(% d. m.)**	**SDF** **(% d. m.)**	**TDF** **(% d. m.)**
Corn grits	1.73 ± 0.00 ^a^	0.63 ± 0.03 ^a^	2.36 ± 0.03 ^a^
Corn + 5% BSG	3.33 ± 0.03 ^c^	1.81 ± 0.05 ^c^	5.13 ± 0.08 ^c^
Corn + 10% BSG	5.72 ± 0.28 ^f^	1.99 ± 0.08 ^c^	7.71 ± 0.21 ^f,g^
Corn + 15% BSG	6.97 ± 0.07 ^h^	2.22 ± 0.23 ^d^	9.19 ± 0.16 ^h^
Corn + 5% SBP	3.19 ± 0.11 ^b,c^	2.37 ± 0.10 ^d,e^	5.56 ± 0.21 ^d^
Corn + 10% SBP	4.28 ± 0.09 ^d^	3.24 ± 0.06 ^f^	7.52 ± 0.15 ^f^
Corn + 15% SBP	6.68 ± 0.11 ^g^	4.33 ± 0.02 ^g^	11.00 ± 0.13 ^i^
Corn + 5% AP	3.00 ± 0.11 ^b^	1.57 ± 0.05 ^b^	4.57 ± 0.05 ^b^
Corn + 10% AP	4.16 ± 0.05 ^d^	1.94 ± 0.05 ^c^	6.10 ± 0.10 ^e^
Corn + 15% AP	5.34 ± 0.07 ^e^	2.53 ± 0.03 ^e^	7.87 ± 0.10 ^g^

Values with different letters in the same column and group of samples (non-extruded, extruded) are significantly different at *p* < 0.05. BSG—brewer’s spent grain, SBP—sugar beet pulp, AP—apple pomace, IDF—insoluble dietary fiber, SDF—soluble dietary fiber, TDF—total dietary fiber. In the samples with BSG and SBP 1% d. m. of pectin was added.

**Table 3:** Starch content, starch damage and pasting properties of non-extruded and extruded samples.

**Sample**	**NON-EXTRUDED**					
	**RS** **(% d. m.)**	**NRS** **(% d. m.)**	**DS** **(%)**	**Peak Viscosity** (BU)	**Viscosity at 92 °C (BU)**	**Viscosity at 50 °C (BU)**
Corn grits	8.01 ± 0.01 ^e^	77.28 ± 0.00 ^f^	1.15 ± 0.10 ^a, b^	566.0 ± 19.8 ^f^	568.5 ± 17.7 ^i^	1027.0 ± 12.7 ^f^
Corn + 5% BSG	9.42 ± 0.16 ^f^	71.24 ± 0.28 ^e^	1.08 ± 0.03 ^a^	415.0 ± 1.4 ^c^	409.0 ± 5.7 ^c^	757.5 ± 3.5 ^c^
Corn + 10% BSG	9.56 ± 0.26 ^f,g^	68.04 ± 0.67 ^d^	1.24 ± 0.04 ^b,c^	315.5 ± 10.6 ^b^	320.5 ± 10.6 ^b^	601.0 ± 15.6 ^b^
Corn + 15% BSG	9.80 ± 0.13 ^g^	64.07 ± 0.71 ^b^	1.17 ± 0.02 ^a,b^	277.0 ± 12.7 ^a^	275.5 ± 9.2 ^a^	516.5 ± 7.8 ^a^
Corn + 5% SBP	8.13 ± 0.08 ^e^	68.42 ± 0.23 ^d^	1.14 ± 0.11 ^a,b^	507.5 ± 10.6 ^e^	512.0 ± 0.0 ^g^	925.0 ± 4.2 ^e^
Corn + 10% SBP	7.50 ± 0.10 ^d^	68.17 ± 0.20 ^d^	1.08 ± 0.06 ^a^	499.0 ± 11.3 ^e^	488.5 ± 4.9 ^f^	927.0 ± 11.3 ^e^
Corn + 15% SBP	6.94 ± 0.01 ^c^	66.23 ± 0.47 ^c^	1.24 ± 0.03 ^b,c^	472.0 ± 9.9 ^d^	462.5 ± 2.1 ^e^	845.5 ± 23.3 ^d^
Corn + 5% AP	6.03 ± 0.16 ^b^	76.73 ± 0.29 ^f^	1.30 ± 0.03 ^c^	547.5 ± 10.6 ^f^	545.0 ± 7.1 ^h^	930.5 ± 9.2 ^e^
Corn + 10% AP	5.94 ± 0.04 ^b^	71.07 ± 0.18 ^e^	1.48 ± 0.01 ^d^	509.5 ± 7.8 ^e^	506.5 ± 9.2 ^f,g^	855.5 ± 4.9 ^d^
Corn + 15% AP	5.49 ± 0.23 ^a^	59.58 ± 0.78 ^a^	1.52 ± 0.08 ^e^	434.5 ± 14.8 ^c^	433.0 ± 15.6 ^d^	747.5 ± 17.7 ^c^
**Sample**	**EXTRUDED**					
	**RS** **(% d. m.)**	**NRS** **(% d. m.)**	**DS** **(%)**	**Peak Viscosity** **(BU)**	**Viscosity at 92 °C (BU)**	**Viscosity at 50 °C (BU)**
Corn grits	0.61 ± 0.00 ^b,c^	83.75 ± 0.39 ^f^	63.28 ± 0.16 ^g^	92.0 ± 7.1 ^b,c^	5.0 ± 7.1 ^a^	123.0 ± 11.3 ^d^
Corn + 5% BSG	0.59 ± 0.00 ^b^	82.37 ± 0.58 ^e^	60.68 ± 0.11 ^e^	88.5 ± 7.8 ^b,c^	0.0 ± 0.0 ^a^	58.5 ± 12.0 ^b^
Corn + 10% BSG	0.98 ± 0.03 ^g^	75.41 ± 0.18 ^b^	55.90 ± 0.48 ^c^	72.0 ± 4.2 ^a,b^	0.0 ± 0.0 ^a^	19.0 ± 7.1 ^a^
Corn + 15% BSG	0.91 ± 0.01 ^f^	73.21 ± 0.74 ^a^	51.30 ± 0.53 ^b^	84.5 ± 6.4 ^b,c^	42.0 ± 8.5 ^c^	98.5 ± 7.8 ^c^
Corn + 5% SBP	0.66 ± 0.01 ^d^	80.08 ± 0.30 ^d^	59.51 ± 0.44 ^d^	130.0 ± 9.9 ^d^	18.5 ± 7.8 ^b^	234.0 ± 11.3 ^g^
Corn + 10% SBP	0.45 ± 0.04 ^a^	78.38 ± 0.48 ^c^	56.33 ± 0.48 ^c^	85.0 ± 14.1 ^b,c^	7.0 ± 9.9 ^a,b^	202.5 ± 13.4 ^f^
Corn + 15% SBP	0.76 ± 0.04 ^e^	75.36 ± 0.25 ^b^	51.69 ± 0.37 ^b^	107.0 ± 33.9 ^c,d^	1.5 ± 2.1 ^a^	169.5 ± 12.0 ^e^
Corn + 5% AP	0.64 ± 0.00 ^c, d^	83.65 ± 0.03 ^f^	61.81 ± 0.56 ^f^	43.5 ± 4.9 ^a^	0.0 ± 0.0 ^a^	0.0 ± 0.0 ^a^
Corn + 10% AP	0.72 ± 0.00 ^e^	78.21 ± 0.86 ^c^	59.62 ± 0.19 ^d^	67.0 ± 7.1 ^a,b^	0.0 ± 0.0 ^a^	0.0 ± 0.0 ^a^
Corn + 15% AP	1.05 ± 0.01 ^h^	74.48 ± 0.52 ^b^	23.05 ± 0.67 ^a^	133.0 ± 5.7 ^d^	0.0 ± 0.0 ^a^	0.0 ± 0.0 ^a^

Values with different letters in the same column and group of samples (non-extruded, extruded) are significantly different at *p* < 0.05. BSG—brewer’s spent grain, SBP—sugar beet pulp, AP—apple pomace, RS—resistant starch, NRS—non-resistant starch, DS—starch damage. In the samples with BSG and SBP 1% d. m. of pectin was added.

**Table 4:** Total phenolic content and antioxidant activity of non-extruded and extruded samples.

**Sample**	**NON-EXTRUDED**	
	**TPC** **(mg GAE/100 g d. m.)**	**AOA** **(% DPPH)**
Corn grits	61.38 ± 1.64 ^b^	17.78 ± 0.03 ^e^
Corn + 5% BSG	65.32 ± 1.35 ^b, c^	16.71 ± 0.06 ^d^
Corn + 10% BSG	69.74 ± 0.79 ^c^	16.08 ± 0.64 ^c,d^
Corn + 15% BSG	71.20 ± 3.77 ^c^	15.41 ± 0.03 ^b,c^
Corn + 5% SBP	53.76 ± 3.72 ^a^	16.51 ± 0.61 ^d^
Corn + 10% SBP	53.30 ± 2.88 ^a^	15.07 ± 0.41 ^b^
Corn + 15% SBP	51.00 ± 0.56 ^a^	13.33 ± 0.40 ^a^
Corn + 5% AP	240.37 ± 2.27 ^d^	24.65 ± 0.07 ^f^
Corn + 10% AP	337.86 ± 2.21 ^e^	31.06 ± 0.60 ^g^
Corn + 15% AP	421.09 ± 3.58 ^f^	38.31 ± 0.27 ^h^
**Sample**	**EXTRUDED**	
	**TPC** **(mg GAE/100 g d. m.)**	**AOA** **(% DPPH)**
Corn grits	48.39 ± 1.06 ^a^	19.51 ± 0.45 ^c^
Corn + 5% BSG	53.32 ± 1.29 ^a,b^	18.85 ± 0.17 ^c^
Corn + 10% BSG	60.10 ± 1.71 ^b,c^	17.36 ± 0.03 ^a,b^
Corn + 15% BSG	63.38 ± 0.62 ^c^	16.97 ± 0.10 ^a^
Corn + 5% SBP	51.33 ± 5.22 ^a^	18.51 ± 0.12 ^b,c^
Corn + 10% SBP	51.04 ± 2.88 ^a^	17.58 ± 0.26 ^a,b^
Corn + 15% SBP	49.90 ± 2.27 ^a^	16.64 ± 0.61 ^a^
Corn + 5% AP	167.18 ± 2.70 ^d^	36.67 ± 0.00 ^d^
Corn + 10% AP	285.36 ± 3.47 ^e^	54.80 ± 0.92 ^e^
Corn + 15% AP	409.13 ± 6.03 ^f^	78.11 ± 1.08 ^f^

Values with different letters in the same column and group of samples (non-extruded, extruded) are significantly different at *p* < 0.05. BSG—brewer’s spent grain, SBP—sugar beet pulp, AP—apple pomace, TPC—total phenolic content, AOA—antioxidant activity. In the samples with BSG and SBP 1% d. m. of pectin was added.

## Data Availability

The data presented in this study are available on request from the corresponding author.
